# Association of obesity with socioeconomic status among adults of ages 18 to 80 years in rural Northwest China

**DOI:** 10.1186/s12889-015-1503-1

**Published:** 2015-02-19

**Authors:** Leilei Pei, Yue Cheng, Yijun Kang, Shuyi Yuan, Hong Yan

**Affiliations:** Department of Epidemiology and Health Statistics, School of Public Health, Xi’an Jiaotong University Health Science Center, No. 76 West Yanta Road, PO Box 46, Xi’an, Shaanxi 710061 PR China; Department of Nutrition and Food Safety Research, School of Public Health, Xi’an Jiaotong University Health Science Center, Xi’an, Shaanxi 710061 PR China

**Keywords:** Obesity, Socioeconomic status, Lifestyles, Disparity

## Abstract

**Background:**

Understanding social disparities in obesity are presently an essential element in establishing public health priorities. However, the association between socioeconomic status (SES) and obesity has not been assessed in rural Northwest China. This study aims to explore the effect of SES on overweight/obesity and abdominal obesity by gender and age in rural Northwest China.

**Methods:**

A total of 3030 participants between the ages of 18 to 80 years from rural Hanzhong, Shaanxi province, Northwest China were enrolled in our study using a two-level stratified random cluster sampling technique. Adjusted odds ratio (AOR) were used to assess the relationship between socioeconomic status and obesity after controlling for confounding factors using logistic regression.

**Results:**

Our results indicated that the prevalence of abdominal obesity (38.8%) was the highest in rural Northwest China when compared with overweight (27.8%) and obesity (5.7%). When adjusting for possible risk factors, there were significant gender disparities in SES-obesity association. In men, the likelihoods of overweight/obesity and abdominal obesity were higher in the high SES groups when compared to the low SES groups. However, women with a high level of education were less likely to have overweight/obesity (AOR:0.78, 95% CI: 0.62, 0.98) than their counterparts with a low level of education. After the inclusion of multiple lifestyle factors, we still observed a strong positive association between age and obesity in the population.

**Conclusions:**

Both gender and age differences in SES-obesity association were clearly observed in our study. Therefore, interventional measures should be employed in rural Northwest China to reduce the obesity epidemic that specifically takes into account gender and age differences.

## Background

Obesity has recently become a major public health problem worldwide and is attributed to high-energy intake or low physical activity levels [1]. In particular, abdominal obesity which is characterized by excessive abdominal fat around the stomach and abdomen, is strongly associated with increased risk of heart disease, hypertension, insulin resistance, type 2 diabetes, asthma, Alzheimer’s Disease as well as various other diseases [2-4]. Globally, approximately 1.5 billion adults were overweight (defined by BMI ≥ 25 kg/m^2^) in 2008, and more than one third of them were obese [5]. Understanding social disparities in health status is presently an important topic on the government health agenda and an essential element in establishing public health priorities. A strong association between socioeconomic status (SES) and obesity has also been widely confirmed [6]. People of low-SES are at greater risk of becoming overweight and obese than people with high SES across all industrial countries, whereas in developing countries people of high-SES people are at increased risk of becoming overweight [7,8]. China is the most populous country in the world, whose total population accounts for one-fifth of the global population. With a large economic development over the past decades, many dramatic changes in lifestyles have occurred among the residents of China. Recent studies suggest that between 1992 and 2002, more than 60 million people in China became obese [9]. However, there is an extreme imbalance in the economic development burdened by different regions of China with lower SES levels found in Northwest areas. Thus, a profound understanding of the SES-obesity relationship in Northwest China can provide meaningful insights for developing effective obesity-prevention and -management programs and policies.

In 2010, we conducted a large investigation on the impact of obesity in rural residents of ages 18 to 80 years in Hanzhong, Shaanxi province in Northwest China. In the present study, our primary objective is to analyze the association between SES and obesity by gender and age among residents in the surveyed areas. To our knowledge, this is the first study to systemically examine the influence of SES on obesity and lifestyles among adults in rural Northwest China.

## Methods

### Study design and participants

A cross-sectional population-based epidemiological and risk factor survey was conducted in 2010 in rural areas of Hanzhong, Shaanxi provinces, in Northwest China. The target population of the survey was adult residents between the ages of 18 to 80 years, who were recruited using a two-level stratified random cluster sampling technique. There were nine townships in the study area and about 17 (15 to 36) villages in each township. Each township was a stratum and one village was randomly chosen from each township. According to local demographic data, all available and eligible adults in the chosen villages were informed and invited to participate in the survey. Hence, it was expected that approximately 3600 participants could be recruited for the survey.

Data for the participants included socio-demographic characteristics and lifestyles, which were obtained by face-to-face interviews with a structured questionnaire. Anthropometric measurements were performed by standard methods using calibrated instruments in an empty room in every village. Standing height was measured using a non-stretchable tape (214 Road Rod™, USA), with the maximum distance from the floor to the highest point on the head facing directly ahead. Participant’s feet were put together without shoes and the arms were placed on either side when the measurements were made. In addition, heels, buttocks and upper back were also in contact with the wall (accurate to 1 mm). Weight was measured using a calibrated electronic scale (Tanita HD-305, Japan) with an accuracy of 0.1 kilograms. Waist circumference (WC; cm) was measured as the minimum circumference between the inferior margin of the ribcage and the crest of the ileum.

Trained field staff from Xi’an Jiaotong University Health Science Center conducted the questionnaire surveys and anthropometric measures. The investigation team consisted of ten to twelve members and one supervisor. During the survey, all field workers were closely monitored by their supervisors and randomly examined. Participants were also re-interviewed when errors and/or missing values were detected. Informed consent was obtained from each participant at the start of the survey. This study was reviewed and approved by the Human Research Ethics Committee of the Xi’an Jiaotong University Health Science Center.

### Research indicators

Body mass index (BMI; kg/m^2^) and waist circumference were used for the assessment of overweight/obesity and abdominal obesity, respectively. In view of the lower obesity-associated metabolism in Chinese people compared to European/North American populations, the Chinese BMI and WC cut-off points proposed by the Working Group on Obesity in China (WGOC) were used to define overweight/obesity and abdominal obesity [10-12]. In this study, overweight and obesity were defined as follows: 24.0 kg/m^2^ ≤ BMI ≤ 27.9 kg/m^2^ (overweight), and BMI ≥ 28.0 kg/m^2^ (obesity) [13]. Abdominal obesity was gender-specific and defined as: WC ≥ 80 cm for women, and WC ≥ 85 cm for men [14]. In order to extend comparisons, different BMI (≥23, ≥24, ≥25, ≥28, ≥30 cm) and WC (≥80, ≥85, ≥88, ≥90, ≥94, ≥102 cm) cut-offs for defining overweight/obesity and abdominal obesity were also adopted.

SES of respondents in surveyed areas was measured by combining the participants mean annual net income per capita and education level, which was then categorized as tertiles indicating the poor, middle and wealthiest income households. The low-SES group was used as a reference and two dummy variables were used to code middle and high SES. Similarly, the age of participants was also classified into tertiles (≤44 years, 45 ~ 54 years and ≥55 years ). According to previous studies [15,16], variables related to the participants’ lifestyles were categorized into two levels, e.g. farming frequency (<3 times/week and ≥3 times/week), hours of TV viewing (<2 h/day and ≥2 h/day), smoking frequency (no smoking and ≥1 cigarette/day), drinking alcohol frequency (no and ≥1/week) as well as amount of vegetable and fruit consumption (<500 g/week and ≥500 g/week).

### Statistical analysis

Data was expressed as numbers or percentages and presented as mean with standard error of the mean. Inter-subgroup differences were examined by Chi-square test and two-tailed t-test. The standardized age prevalence of overweight/obesity and abdominal obesity was calculated by a direct method using 2010 China population census data as the standard population. For assessing the effects of annual net income per capita and education level on overweight/obesity and abdominal obesity for participants by gender and age, an adjusted odds ratio (AOR) was obtained by a multivariate logistic regression analysis after controlling for socio-demographic characteristics and risk factors. The SPSS 13.0 (SPSS Inc., Chicago, Illinois, USA) was used for all statistical analysis and significance was achieved from statistical tests when *P* < 0.05.

## Results

### Participant’s baseline characteristics

A total of 3030 eligible participants between the ages of 18 to 80 years were included from surveyed areas, with a response rate of 84.17% (Table 1). The mean age amongst participants was 50.04 ± 11.80 years, with women accounting for 65.3% of the total number of participants. The average number of years of education for the participants was 6.77 ± 3.72 years (range 0–18 years). Among the participants, 28.4% were from poor households, 32.4% were from middle households, while 39.2% were from wealthy households based on the annual net income per capita. The mean BMI and WC were significantly higher in men than women (P < 0.05). Gender differences were also observed in the rural Hanzhong participants’ in terms of age, education years as well as employment, income and marriage status (*P* < 0.001). With respect to the participants’ lifestyle, our study revealed that women had a significantly higher frequency for farming, but lower alcohol consumption, hours of TV viewing and smoking frequency than men.

**Table 1:** **Socio-demographic characteristics and lifestyles of enrolled adult participants at the age of 18 to 80 years in rural Hanzhong, Shaanxi province in 2010** ^**a**^

**Socio-demographic variables** ^**b**^	**Female**	**Male**	**Total**
Body mass index (BMI; kg/m^2^)*	22.91(3.22)	22.95 (5.52)	22.92 (4.17)
Waist circumference (WC; cm)*	78.08 (8.64)	81.37 (9.12)	79.22 (8.95)
Age (years)*			
≤44	664 (33.8)	317 (30.3)	981 (32.6)
45 ~ 54	674 (34.3)	315 (30.1)	989 (32.9)
≥55	626 (31.9)	414 (39.6)	1040 (34.6)
Marriage status*			
Married	1803 (98.7)	955 (95.7)	2758 (97.6)
Single/divorced/widowed	24 (1.3)	43 (4.3)	67 (2.4)
Employment status*			
Farming	1787 (90.7)	807 (77.3)	420 (13.9)
No farming	183 (9.3)	237 (22.7)	2594 (86.1)
Level of education*			
Low	627 (32.0)	177 (16.9)	804 (26.7)
Middle	426 (21.7)	228 (21.8)	654 (21.7)
High	908 (46.3)	642 (61.3)	1550 (51.5)
Annual net income per capita*			
Low	517 (26.9)	323 (31.2)	840 (28.4)
middle	659 (34.3)	299 (28.9)	958 (32.4)
High	745 (38.8)	412 (39.8)	1157 (39.2)
*Lifestyles* ^*c*^			
Farming frequency*			
<3 times/week	677 (34.7)	408 (39.5)	1085 (35.8)
≥3 times/week	1274 (65.3)	624 (60.5)	1898 (63.6)
Hours of TV viewing*			
<2 h/day	756 (40.1)	311 (30.3)	1067 (36.7)
≥2 h/day	1128 (59.9)	716 (69.6)	1844 (63.3)
Alcohol consumption*			
0 times/week	1902 (97.8)	842 (81.7)	2744 (92.2)
≥1 times/week	43 (2.2)	188 (18.3)	231 (7.8)
Smoking frequency*			
0 cigarette/day	1925 (99.2)	379 (36.4)	2304 (77.2)
≥1 cigarette/day	16 (0.8)	663 (63.6)	679 (22.8)
Vegetable and fruit consumption			
<500 g/week	1411 (73.0)	761 (74.2)	2172 (73.5)
≥500 g/week	521 (27.0)	264 (25.8)	785 (26.5)
Total	1978	1052	3030

^a^Values are given as mean (SD) or the number (percentage) of the study population across socio-demographic characteristics by gender.

^b^Missing values for socio-demographic characteristics: 16 for Employment status, 75 for income, 22 for education, 205 for marriage, 20 for age.

^c^Missing values for lifestyles: 73 for Vegetable and fruit consumption, 47 for Farming frequency and Smoking frequency, 55 for Alcohol consumption, 119 for Hours of TV viewing.

*Denotes P < 0.05 for differences in socio-demographic characteristics and lifestyles by gender.

### Prevalence of overweight/obesity and abdominal obesity in rural Northwest China

Based on BMI and WC cut-offs standards associated with the Chinese population, the prevalence of overweight, obesity and abdominal obesity was 27.8%, 5.7% and 38.8%, respectively, in rural Hanzhong, Shaanxi province. The age-standardized percentage of overweight and obesity was 23.4% and 5.9%, respectively (24.7% (overweight) and 7.5% (obesity) in men and 22.8% (overweight) and 4.5% (obesity) in women). The age-standardized percentage of abdominal obesity was 32.5% (31.1% in men and 31.5% in women).

Our results revealed that there is a discrepancy in the prevalence of overweight, obesity and abdominal obesity across different socio-demographic groups and lifestyles as shown in Table 2. Among female participants, the prevalence of obesity increased with age. There was also a significant difference in the prevalence of obesity across education levels and lifestyles, such as farming frequency as well as vegetable and fruit consumption. In men, a higher prevalence of overweight and abdominal obesity was observed with a higher income and level of education. Marriage and employment status was also related to obesity in men. Amongst men with low farming activity frequency and smoking frequency as well as high vegetable and fruit intake, there was a high prevalence of obesity.

**Table 2:** **Prevalence of overweight, obesity and abdominal obesity among the study population across different socio-demographic characteristics and lifestyles by gender in rural Hanzhong, Shaanxi province in 2010** ^**a**^

	**Overweight**			**Obesity**			**Abdominal obesity**		
	**(24.0 kg/m** ^**2**^ **≤ BMI ≤ 27.9 kg/m** ^**2**^ **)**			**(BMI ≥ 28 kg/m** ^**2**^ **)**			**(WC ≥ 80 cm for female and ≥85 cm for male)**		
**Socio-demographic characteristics**	**Female**	**Male**	**Total**	**Female**	**Male**	**Total**	**Female**	**Male**	**Total**
Age									
≤44	173(26.1)†	115(36.3)	288(29.4^)‡^	24(3.6)^†^	22(6.9)	46(4.7)^)‡^	207(31.2)^†^	104(32.8)	311(31.7)^)‡^
45 ~ 54	249(36.9)	114(36.2)	363(36.7)	36(5.3)	18(5.7)	54(5.5)	295(43.8)	123(39.0)	418(42.3)
≥55	223(35.6)	126(30.4)	349(33.6)	50(8.0)	23(5.6)	73(7.0)	300(48.1)	139(33.6)	439(42.3)
Marriage status									
Married	593(32.9)	337(35.3)*	930(33.7)^)‡^	100(5.5)	60(6.3)	160(5.8)	731(40.5)	342(35.8)*	1073(38.9)^)‡^
Single/divorced/widowed	5(20.8)	5(11.6)	10(14.9)	1(4.2)	2(4.7)	3(4.5)	7(29.2)	5(11.6)	12(17.9)
Employment status									
Farming	592(33.1)	259(32.1)*	851(32.8)	100(5.6)	42(5.2)*	142(5.5)	741(41.5)	263(32.6)*	1004(38.7)
No farming	57(31.1)	97(40.9)	154(36.7)	10(5.5)	21(8.9)	31(7.4)	64(35.0)	102(43.0)	166(39.5)
Level of education									
Low	220(35.1)	39(22.0)*	259(32.2)	42(6.7)	7(4.0)	49(6.1)	287(45.8)†	52(29.4)*	339(42.2)
Middle	147(34.5)	75(32.9)	222(33.9))	29(6.8)	17(7.5)	46(7.0)	181(42.5)	70(30.7)	251(38.4)
High	279(30.7)	242(37.7)	521(33.6)	39(4.3)	39(6.1)	78(5.0))	335(37.0)	244(38.0)	579(37.4)
Annual net income per capita									
Low	152(29.4)	84(26.0)*	236(28.1)^)‡^	30(5.8)	15(4.6)	45(5.4)	210(40.7)	94(29.1)*	259(32.2)
Middle	221(33.5)	113(37.8)	334(34.9)	39(5.9)	24(8.0)	63(6.6)	271(41.2)	120(40.1)	222(33.9)
High	261(35.0)	156(37.9)	417(36.0)	36(4.8)	24(5.8)	60(5.2)	304(40.8)	150(36.4)	521(33.6)
*Lifestyles*									
Farming frequency									
<3 times/week	236(34.9)	168(41.2)*	404(37.2)^)‡^	51(7.5)^†^	34(8.3)*	85(7.8)^)‡^	291 (43.0)	163(40.0)*	454(41.9)^)‡^
≥3 times/week	407(31.9)	182(29.2)	589(31.0)	59(4.6)	28(4.5)	87(4.6)	508(39.9)	197(31.6)	705(37.2)
Hours of TV viewing									
<2 h/day	237(31.3)	93(29.9)	330(30.9)^)‡^	45(6.0)	19(6.1)	64(6.0)	313(41.5)	102(32.8)	415(38.9)
≥2 h/day	380(33.7)	258(36.0)	638(34.6)	58(5.1)	43(6.0)	101(5.5)	456(40.5)	259(36.2)	715(38.8)
Alcohol consumption									
0 times/week	630(33.1)	285(33.8)	915(33.3)	110(5.8)	53(6.3)	163(5.9)	779(41.0)	292(34.7)	1071(39.0)
≥1 times/week	11(25.6)	67(35.6)	78(33.8)	0(0.0)	9(4.8)	9(3.9)	17(39.5)	70(37.2)	87(37.7)
Smoking frequency									
0 cigarette/day	635(33.0)	153(40.4)*	788(34.2)	107(5.6)	25(6.6)	132(5.7)	787(40.9)	151(39.8)*	938(40.7)^)‡^
≥1 cigarette/day	5(31.3)	202(30.5)	207(30.5)	2(12.5)	38(5.7)	40(5.9)	7(43.8)	215(32.4)	222(32.7)
Vegetable and fruit consumption									
<500 g/week	446(31.6)*	252(33.1)	698(32.1)^)‡^	83(5.9)	43(5.7)	126(5.8)	561(39.8)*	254(33.4)*	815(37.5)^)‡^
≥500 g/week	188(36.1)	99(37.5)	287(36.6)	26(5.0)	19(7.2)	45(5.7)	229(44.0)	107(40.5)	336(42.8)
Total	650(32.9)	356(33.8)	1003(33.2)	63(6.0)	110(5.6)	173(5.7)	366(34.8)*	808(40.9)	1174(38.8)

^a^Values represent the number of overweight, obesity and abdominal obesity and the prevalence is included in the bracket.

*Denote P < 0.05, suggesting a significant difference in overweight, obesity and abdominal obesity by socio-demographic characteristics and lifestyles among male adults.

^†^Denote P < 0.05 for prevalence of overweight, obesity and abdominal obesity across socio-demographic characteristics and lifestyles among female adults.

^‡^Denote P < 0.05 for comparison across different socio-demographic characteristics and lifestyles in total population.

BMI percentage cutoffs of ≥23, ≥24, ≥25, ≥28, ≥30 kg/m^2^ among the participants in rural Hanzhong were as follows: 45.5%, 33.2%, 22.7%, 5.7% and 1.7% respectively. By gender, the BMI percentages cutoffs of ≥23, ≥24, ≥25, ≥28 and ≥30 kg/m^2^ in study areas were 46.0%, 32.9%, 21.6%, 5.6% and 1.5%, respectively, for women and 44.5%, 33.8%, 24.7%, 6.0% and 2.0%, respectively, for men. WC percentage cutoffs of ≥80, ≥85, ≥88, ≥90, ≥94 and ≥102 cm were 40.9%, 22.1%, 13.7%, 9.6%, 4.7% and 0.8%, respectively for women and 54.3%, 34.8%, 23.8%, 19.4%, 11.2% and 2.1%, respectively for men.

### The association of obesity with SES in rural Northwest China

As shown in Table 3, there is a statistically significant correlation between obesity and SES associated with gender amongst the participants in rural Hanzhong, when adjusting for socio-demographic and risk factors analyzed in the study and using a logistic regression model. It could be found that the association between SES and obesity was statistically significant only when BMI was used for the female population, meanwhile a significant association was observed in males for both parameters (BMI and WC). For example, women with a high level of education were less likely to have overweight/obesity (AOR:0.78, 95% CI: 0.62, 0.98) than their counterparts with a low level of education. However, amongst men, it was noted that a higher risk of overweight/obesity was found in higher education (AOR:2.26, 95% CI: 1.50, 3.42) or income group (AOR: 1.74,95% CI: 1.25,2.42) when compared to the low education or income group. Men with a high level of education (AOR:1.64, 95% CI: 1.12, 2.41) or income (AOR:1.52, 95% CI: 1.10, 2.11) were also more likely to have abdominal obesity than their counterparts with a low level of education or income. The results above suggested that there was a clear gender difference in the association between SES and overweight/obesity. Furthermore, being a woman, the person had a lower risk of being overweight/obese (AOR: 0.77, 95% CI: 0.60, 0.98) compared to a man.

**Table 3:** **The association between obesity and SES among enrolled adult participants aged 18–80 years by gender and age groups in rural Hanzhong, Shaanxi province in 2010** ^**ab**^

***Class differences***	**Abdominal obesity**	**Overweight/obesity**
	**(WC ≥ 85 cm for male and WC ≥ 80 cm for female)**	**(BMI ≥ 24 kg/m** ^**2**^ **)**
*Female*		
Annual net income per capita		
Medium	1.23 (0.97, 1.57)	1.38 (1.06, 1.78)*
High	1.22 (0.96, 1.56)	1.49 (1.16, 1.92)*
Level of education		
Medium	0.89 (0.69, 1.14)	0.94 (0.72, 1.23)
High	0.69 (0.56, 0.86)*	0.78 (0.62, 0.98)*
*Male*		
Annual net income per capita		
Medium	1.75 (1.24, 2.46)*	1.74 (1.23, 2.47)*
High	1.52 (1.10, 2.11)*	1.74 (1.25, 2.42)*
Level of education		
Medium	1.13 (0.73, 1.74)	1.81 (1.14, 2.86)*
High	1.64 (1.12, 2.41)*	2.26 (1.50, 3.42)*
*Gender differences*		
Men	1.00	1.00
Women	1.08 (0.91, 1.28)	0.77 (0.60, 0.98)*
*The youngest participants*		
Annual net income per capita		
Medium	1.26 (0.81, 1.94)	1.32 (0.84, 2.08)
High	1.35 (0.88, 2.07)	1.50 (0.97, 2.34)
Level of education		
Medium	1.36 (0.79, 2.35)	2.14 (1.16, 3.94)*
High	1.14 (0.69, 1.90)	1.80 (1.01, 3.19)*
*The oldest participants*		
Annual net income per capita		
Medium	1.76 (1.29, 2.42)*	1.67 (1.20, 2.30)*
High	1.18 (0.87, 1.60)	1.29 (0.94, 1.77)
Level of education		
Medium	0.88 (0.64, 1.20)	1.01 (0.73, 1.41)
High	0.96 (0.73, 1.31)	1.19 (0.86, 1.65)
*Difference by age groups*		
The youngest participants	1.00	1.00
The medium participants	1.58 (1.31, 1.90)*	1.40 (1.16, 1.69)*
The oldest participants	1.58 (1.32, 1.90)*	1.22 (1.01, 1.47)*

^a^The stratified analysis was conducted for all participants and separate multivariate logistic models were fit for each gender groups and age groups when socio-demographic factors and risk factors were adjusted for. The low-SES group was treated as the reference group.

^b^Values were given as adjusted odds ratio (95% CI) unless otherwise stated.

*Denote P < 0.05.

An analysis of SES-obesity association stratified by age groups was also performed after adjusting for socio-demographic and risk factors analyzed in the study (Table 3). Among the youngest participants, we found that participants of medium (AOR:2.14, 95% CI: 1.16, 3.94) and high (AOR:1.80, 95% CI: 1.01, 3.19) education level had a higher likelihood of overweight/obesity than those in the low-education group. Among the elderly participants, we also observed that participants with a medium income were more likely to be abdominally obese (AOR:1.76, 95% CI: 1.29, 2.42) and overweight/obese (AOR:1.67, 95% CI: 1.20, 2.30) in contrast with those with a low-income. After controlling for socio-demographic and risk factors, a strongly positive association was still observed between age and obesity in the participants.

### The relationship between lifestyle and SES

When adjusting for socio-demographic factors, an association between lifestyle and SES was also observed by gender, when using low-SES as the reference group (Table 4). Our study showed that the SES disparities in lifestyles including farming frequency, TV viewing as well as vegetable and fruit consumption was pronounced for women. Among female participants in surveyed areas, the duration of TV viewing as well as amount of vegetable and fruit consumption were found to increase with income and education level, whereas farming frequency was just the opposite. Among men, farming frequency also decreased with the increment of income and education level, while duration of TV viewing as well as amount of vegetable and fruit consumption increased with income and education level. Women reported lower duration of TV viewing, alcohol consumption and smoking frequency than men, but displayed increased farming frequency. Furthermore, SES disparities in lifestyles across the youngest and oldest groups were also observed and shown in the Table 4. In the youngest participants, a positive association was clearly observed between amount of vegetable and fruit consumption and increased income. In addition, duration of TV viewing was much more in participants of medium and high education than that of low education. There was also an inverse correlation between farming frequency and SES. Among the oldest participants, on the whole, consumption of vegetable and fruit, alcohol intake, smoking frequency and duration of TV viewing were positively associated with SES. Furthermore, it was evident that the oldest participants had less duration of TV viewing, alcohol consumption as well as consumption of vegetable and fruit, as well as did more farm work.

**Table 4:** **Adjusted odds ratio (AOR) and 95% CI of risk factors for obesity across socioeconomic status (SES) by gender and age groups among enrolled adult participants of ages 18 to 80 years in rural Hanzhong, Shaanxi province in 2010** ^**ab**^

**Class differences**	**Farming frequency**	**TV viewing**	**Alcohol consumption**	**Smoking frequency**	**Vegetable and fruit consumption**
*Female*					
Annual net income per capita					
Medium	0.93 (0.71, 1.21)	1.27 (0.99, 1.65)*	1.89 (0.76, 4.69)	0.74 (0.18, 3.11)	1.52 (1.12, 2.06)*
High	0.60 (0.46, 0.77)*	1.30 (1.01, 1.67)	1.40 (0.54, 3.59)	1.02 (0.26, 3.93)	2.18 (1.63, 2.93)*
Level of education					
Medium	1.08 (0.82, 1.43)	1.13 (0.86, 1.47)	1.01 (0.41, 2.47)	0.15 (0.02, 1.29)	1.12 (0.81, 1.56)*
High	0.59 (0.46, 0.76)*	1.88 (1.48, 2.40)*	0.76 (0.33, 1.75)	0.48 (0.15, 1.56)	1.47 (1.11, 1.95)*
*Male*					
Annual net income per capita					
Medium	1.13 (0.80, 1.60)	1.47 (1.03, 2.09)*	1.14 (0.73, 1.79)	1.21 (0.87, 1.70)	1.29 (0.86, 1.93)
High	0.60 (0.44, 0.82)*	1.37 (0.99, 1.90)	1.49 (0.99, 2.24)	1.25 (0.91, 1.71)	2.27 (1.58, 3.25)*
Level of education					
Medium	1.07 (0.68, 1.67)	1.10 (0.71, 1.69)	1.56 (0.90, 2.72)	1.22 (0.79, 1.86)	1.30 (0.76, 2.22)
High	0.53 (0.36, 0.77)*	1.73 (1.20, 2.51)*	1.42 (0.87, 2.31)	1.12 (0.78, 1.61)	2.16 (1.38, 3.38)*
*Gender differences*					
Men	1.00	1.00	1.00	1.00	1.00
Women	1.22 (1.03, 1.44)*	0.71 (0.60, 0.85)*	0.10 (0.07, 0.14)*	0.003 (0.002, 0.006)*	1.19 (0.99, 1.42)
*The youngest participants*					
Annual net income per capita					
Medium	0.60 (0.39, 0.93)*	1.32 (0.86, 2.02)	0.80 (0.37, 1.68)	1.28 (0.65, 2.53)	1.17 (0.75, 1.81)
High	0.36 (0.23, 0.54)*	1.42 (0.94, 2.15)	1.48 (0.75, 2.90)	1.27 (0.67, 2.42)	1.72 (1.13, 2.62)*
Level of education					
Medium	0.89 (0.51, 1.57)	1.70 (1.01, 2.85)*	2.05 (0.72, 5.87)	0.89 (0.29, 2.71)	0.89 (0.52, 1.53)
High	0.36 (0.22, 0.60)*	2.11 (1.32, 3.35)*	1.08 (0.40, 2.94)	0.53 (0.19, 1.46)	1.34 (0.83, 2.17)
*The oldest participants*					
Annual net income per capita					
Medium	1.16 (0.84, 1.60)	1.51 (1.10, 2.09)*	2.20 (1.13, 4.27)*	1.67 (1.03, 2.70)*	1.69 (1.12, 2.56)*
High	0.94 (0.69, 1.27)	1.37 (1.00, 1.87)*	1.39 (0.69, 2.80)	1.42 (0.90, 2.24)	2.23 (1.52, 3.27)*
Level of education					
Medium	1.13 (0.82, 1.57)	1.12 (0.82, 1.53)	0.82 (0.40, 1.72)	0.70 (0.43, 1.15)	1.51 (1.00, 2.29)
High	0.57 (0.41, 0.79)*	1.77 (1.27, 2.46)*	1.05 (0.54, 2.03)	0.65 (0.41, 1.03)	2.44 (1.64, 3.64)*
*Difference by age groups*					
The youngest participants	1.00	1.00	1.00	1.00	1.00
The medium participants	1.91 (1.58, 2.31)*	0.74 (0.61, 0.90)*	0.88 (0.64, 1.20)	1.09 (0.89, 1.35)	0.78 (0.65, 0.95)*
The oldest participants	1.27 (1.07, 1.52)*	0.39 (0.32, 0.47)*	0.55 (0.39, 0.78)*	0.94 (0.76, 1.17)	0.43 (0.35, 0.53)*

^a^The stratified analysis was conducted for all participants and separate logistic models were fit for each gender groups and age groups when socio-demographic factors were adjusted for. The low-SES group was treated as the reference group.

^b^Values were given as adjusted odds ratio (95%CI) unless otherwise stated.

*Denote P < 0.05.

## Discussion

In this study, we examined the prevalence of overweight/obesity and abdominal obesity as well as their associations with SES in rural Hanzhong, Northwest China in 2010. Our results indicated that the prevalence of abdominal obesity was higher in rural Northwest China when compared with overweight or obesity. Overall, it also appears that there is a significant difference in the SES-obesity association by gender in rural Northwest China. Additionally, the relationship between SES and the participants’ lifestyle may be an important influencing factor that warrants further exploration.

Results indicated that the crude prevalence of overweight and obesity was 33.2% (age-standardized percentage 28.9%) and 5.7% (age-standardized percentage 5.3%), respectively, among participants in rural Hanzhong, Northwest China in 2010. The figures reported from other studies conducted in China have varied considerably. For instance, approximately 37.5% of adults in Zhejiang province in 2009 were reported to be overweight/obese (BMI ≥ 24 kg/m^2^, age-standardized rate 36.4%) [17]. A study performed in Shanghai during 2007–2008 reported the prevalence of overweight/obesity (BMI ≥ 24 kg/m^2^) to be 43.4% [18]. A 2002 China Health and Nutrition survey indicated that 37.9% of adults aged ≥ 18 years were overweight/obese (BMI ≥ 24 kg/m^2^) nationwide [19]. Globally, it was estimated that the prevalence of obesity (BMI ≥ 30 kg/m^2^) was the lowest in south Asia in both men (1.4%) and women (2.9%) [20]. By comparison, it seems that the prevalence of overweight/obesity observed in our study population is approaching the Chinese mean level as well as the values reported in eastern China, which are also much higher than that reported in south Asia.

Our study further showed that the age-standardized prevalence of abdominal obesity had reached 33.5% for men and 37.5% for women, respectively. These results were consistent with other reported studies in China [17,18]. Data available from the Chinese Center for Disease Control and Prevention (CDC) estimated that the average prevalence of abdominal obesity was about 30.4% nationwide in 2004 [21]. In a 2007–2008 survey in Shanghai, there was a report of 22.4% of abdominal obesity among the participants (WC ≥ 90 cm for men with 27.3% and WC ≥ 85 cm for women with 17.1%) [18]. Data from the Zhengjiang Province indicated that age-and-sex adjusted abdominal overweight and obesity rates were 32.2% and 12.3%, respectively [17]. It is apparent from these findings that the abdominal obesity epidemic is more severe relative to the national average level, and lower than that of eastern China.

In exhaustive reviews by Sobal and Mclaren, it was concluded that the relationship between SES and obesity had two patterns: the “developing country pattern” and “developed country pattern”. As one moved from high- to medium- to low-income countries, the proportion of positive SES-obesity associations increased and the proportion of negative associations decreased, for both men and women [22,23]. When adjusting for socio-demographic factors and possible risk factors, we found there were significant disparities in SES-obesity association between genders when measured by different SES indicators. In men, the prevalence of overweight/obesity and abdominal obesity was higher in the higher income and education level groups. In women, overweight/obesity was less likely to occur in the higher education level groups when compared to low education groups, suggesting that the social patterning of weight-related attributes of women was perhaps in transition across the developmental spectrum. In addition, a higher likelihood of overweight/obesity in men was found relative to women as determined by our study. Such atypical SES-obesity relationships in our study were found to be similar to other studies in developing countries such as Thailand and the Philippines [24,25], indicating that the transition of SES-obesity association in women was comparatively faster than that in men. Monteiro et al. also found a similar transition, highlighting a shift of obesity towards persons with low SES as a country’s annual gross national product increases [26].

Studies had shown that there might be SES differences in dietary intake, participation in physical exertion both at work and during leisure time, exposure to early life under-nutrition or over-nutrition, exposure to social or psychological stress, and exposure to health education and health-care [26-28]. These factors might further differ between the sexes, for example, adult women often have less secure and well-paid employment than men, and are both more at risk of poverty, potentially giving them less control over access to nutritious food [1]. However, gender differences associated between SES and obesity are complex and may be confounded by other factors such as health behaviors and socio-cultural norms [29,30]. Previous research concluded that “habitus theory” plays a vital role in gender-related differences [31]. Generally speaking, in women, a thinner body is socially valued and such opinion may be dictated by social pressures on highly educated women to be thin than highly educated men [32]. For men, a larger body size is likely to be valued as a sign of physical dominance and prowess [33]. With men being the traditional wage earners in families, it is plausible that income and pursuit of physical dominance remain linked. Thus it is not surprising to find a faster transition for SES-obesity relationship in women than men.

To further explore gender disparities in the SES-obesity relationship, we analyzed the correlation between SES and specific lifestyles associated with obesity by gender among the participants after adjusting for socio-demographic factors. Overall, duration of TV viewing was positively associated with SES based on education level and income in both genders. Nevertheless, farming frequency decreased with the SES of participants. In our study, it was also observed that women had less duration of TV viewing and alcohol consumption, but they did take on much more farming work than men. In order to bring in more money and supplement farming income, most men leave the village to work temporary jobs in urban areas, while more women stay at home and occupy themselves with farming. Due to the low economic level in the surveyed areas, television viewing was considered as the primary leisure after work among local participants, especially in the high-SES groups. Thus, lifestyle differences by gender may contribute to the sex disparity observed in the SES-obesity relationship.

In the present analyses, the age effect was strong for both sexes: obesity rose with age. It should be noted that duration of TV viewing, alcohol consumption as well as vegetable and fruit consumption were lower in the oldest participants compared to the youngest participants, while farming frequency was just the opposite. However, after the inclusion of multiple lifestyle factors, we still noted a strong positive association between age and obesity in this population, which indicated the lifestyle factors have limited effect on age-obesity association. This is in line with a previous study performed in Thailand [25].

Several limitations of this study should be noted. First, due to the cross-sectional design of our study, we cannot draw any cause-effect conclusion based on the study results. Second, as the study is confined to rural Northwest China, the conclusions from this study may not be generalizable to other areas in China. Third, anthropometric indicators such as height, weight, and WC were measured directly but information on lifestyles were self-reported. Thus, the possibility of information bias should be considered. Despite these limitations, this is the first study to determine the relationship between SES and obesity in rural Northwest China, which provides important clues for prevention and control of local obesity.

## Conclusions

In summary, there is an obesity epidemic in rural Northwest China, which is increasingly approaching the national average level. On the whole, the prevalence of obesity were higher in men than in women, and positively associated with age. The gender difference in SES-obesity association was clearly observed, suggesting that the transition of SES-obesity association from “developing country pattern” into “developed country pattern” in women was comparatively faster than that in men. After adjusting for multiple lifestyle factors, we still discerned a strong positive age-obesity association in the population. Therefore, it is necessary and crucial to implement interventional-based strategies focused on gender and age to reduce the obesity epidemic in rural Northwest China.
